# Anticancer drugs for the modulation of endoplasmic reticulum stress and oxidative stress

**DOI:** 10.1007/s13277-015-3797-0

**Published:** 2015-07-19

**Authors:** Ammad Ahmad Farooqi, Kun-Tzu Li, Sundas Fayyaz, Yung-Ting Chang, Muhammad Ismail, Chih-Chuang Liaw, Shyng-Shiou F. Yuan, Jen-Yang Tang, Hsueh-Wei Chang

**Affiliations:** 1 Institute of Biomedical and Genetic Engineering (IBGE), KRL Hospital, Islamabad, Pakistan; 2 Department of Biomedical Science and Environmental Biology, Kaohsiung Medical University, Kaohsiung, Taiwan; 3 Laboratory for Translational Oncology and Personalized Medicine, RLMC, Lahore, Pakistan; 4 Doctor Degree Program in Marine Biotechnology, National Sun Yat-sen University/Academia Sinica, Kaohsiung, Taiwan; 5 Department of Marine Biotechnology and Resources, National Sun Yat-sen University, Kaohsiung, Taiwan; 6 Translational Research Center, Kaohsiung Medical University Hospital, Kaohsiung Medical University, Kaohsiung, Taiwan; 7 Cancer Center, Kaohsiung Medical University Hospital; Kaohsiung Medical University, Kaohsiung, Taiwan; 8 Department of Radiation Oncology, Faculty of Medicine, College of Medicine, Kaohsiung Medical University, Kaohsiung, Taiwan; 9 Department of Radiation Oncology, Kaohsiung Medical University Hospital, Kaohsiung, Taiwan; 10 Department of Radiation Oncology, Kaohsiung Municipal Ta-Tung Hospital, Kaohsiung, Taiwan; 11 Institute of Medical Science and Technology, National Sun Yat-sen University, Kaohsiung, Taiwan; 12 Research Center of Environmental Medicine, Kaohsiung Medical University, Kaohsiung, Taiwan

**Keywords:** Anticancer, ER stress, ROS, Natural products

## Abstract

Prior research has demonstrated how the endoplasmic reticulum (ER) functions as a multifunctional organelle and as a well-orchestrated protein-folding unit. It consists of sensors which detect stress-induced unfolded/misfolded proteins and it is the place where protein folding is catalyzed with chaperones. During this folding process, an immaculate disulfide bond formation requires an oxidized environment provided by the ER. Protein folding and the generation of reactive oxygen species (ROS) as a protein oxidative byproduct in ER are crosslinked. An ER stress-induced response also mediates the expression of the apoptosis-associated gene C/EBP-homologous protein (CHOP) and death receptor 5 (DR5). ER stress induces the upregulation of tumor necrosis factor-related apoptosis inducing ligand (TRAIL) receptor and opening new horizons for therapeutic research. These findings can be used to maximize TRAIL-induced apoptosis in xenografted mice. This review summarizes the current understanding of the interplay between ER stress and ROS. We also discuss how damage-associated molecular patterns (DAMPs) function as modulators of immunogenic cell death and how natural products and drugs have shown potential in regulating ER stress and ROS in different cancer cell lines. Drugs as inducers and inhibitors of ROS modulation may respectively exert inducible and inhibitory effects on ER stress and unfolded protein response (UPR). Reconceptualization of the molecular crosstalk among ROS modulating effectors, ER stress, and DAMPs will lead to advances in anticancer therapy.

## Introduction

### ER stress

Endoplasmic reticulum (ER) provides suitable folding to generate functional proteins. When the cellular environment changes or cells are affected by DNA damage and oxidative stress, misfolding proteins may accumulate in the ER leading to increased ER stress. In response to ER stress, cells activate a series of signaling proteins called *unfolded protein response* (UPR) which favors suitable ER protein folding [1]. Both ER stress and UPR activation are commonly reported in many different cancers. Information obtained from high throughput technologies has substantially improved our understanding of the UPR. This particularly holds for stress sensors that balance ER homeostasis in the protection of cell viability for mild ER stress [2] or leads to intrinsic mitochondrial apoptosis [3] for severe ER stress [4].

Rapidly emerging evidence highlight the key roles of versatile regulators, particularly inositol-requiring protein 1α (IRE1α), protein kinase RNA-like endoplasmic reticulum kinase (PERK), and activating transcription factor 6 (ATF6) in transducing information from the ER to the cytosol and nucleus to mediate biological activities [1, 2, 5, 6]. It is known that immunoglobulin-heavy-chain-binding protein (GRP78/BIP)-bound stress sensors remain inactive and unfolded protein accumulations in the ER induce the activation of ATF6, IRE1α, and PERK [7]. Unbinding GRP78 from ATF6 exposes Golgi-localization sequence (GLS) within ATF6 [8] to guide the protein to Golgi by interacting with the coat protein II (COPII) complex [9], and within Golgi, it undergoes proteolytic processing by site-1 protease (S1P) and site-1 protease (S2P) [10]. The proteolytically processed ATF6 fragment (ATF6f) acts as a transcription factor and moves into the nucleus to transcriptionally upregulate target genes, including GRP78, C/EBP-homologous protein (CHOP), and X-box binding protein 1 (XBP1) [1, 11]. Unbinding of GRP78 from IRE1 induced homodimer formation and the activation of IRE1 through autophosphorylation [12]. Phospho-IRE1 excises a 26-bp fragment from unspliced XBP1 messenger RNA (mRNA) to form spliced XBP1s mRNA after re-ligation [13]. Nuclear accumulation of XBP1 protein follows binding to UPR elements (UPREs) to trigger target genes. PERK-induced phosphorylation of phospho-eukaryotic initiation factor-alpha (eIF2α) results in translational inhibition [14]. However, ATF4 mRNA escapes eIF2α-mediated translational suppression [15]. ATF4 transcriptionally upregulated CHOP and protein phosphatase 1 regulatory subunit 15A (PPP1R15A; GADD34) [16]. eIF2α dephosphorylation was triggered by GADD34-bound protein phosphatase 1C (PP1C) [17]. Next, we discuss another widely studied mechanism of cellular oxidative stress in ER.

### Oxidative stress

The biology of free radical generation has attracted considerable scientific interest, and we now categorically know that two mechanisms mediate the generation of reactive oxygen species (ROS). Oxidative folding machinery induced by UPR in the ER and mitochondria is associated with free radical generation. Both ROS and reactive nitrogen species (RNS) are generated in response to different cellular stresses and as byproducts of normal cellular metabolism [18]. ROS and RNS have opposite roles at varying concentrations. For example, high concentrations of these species induced cellular damage but was reported to be advantageous at low/moderate concentrations while working synchronously with cellular antioxidant defense mechanisms which detect, respond to, and transmit these signals to maintain cellular redox homeostasis [19]. In addition, NADPH oxidases (NOX) are responsible for ROS generation. The modulation of NADPH oxidases by natural products may change the ROS level [20].

Oxidative stress is a condition in which ROS is overproduced and cannot be balanced by the available antioxidant machinery. Mitochondria are the major production sites of the superoxide anion ozone (triplet stage molecular oxygen) that later forms secondary species, namely hydroxyl radical, hydrogen peroxide, hydroperoxyl radical, and hypochlorous acid [21]. Proper folding of proteins is a critical and multistep process and requires an oxidizing–folding environment. This particularly sensitive procedure is ROS dependent and occurs in the ER where disulfide bond formation takes place during the folding process. For example, the ER membrane-associated oxidoreductin (ERO-1) uses a flavin adenine dinucleotide (FAD)-dependent procedure to transfer electrons from the 58-kDa protein disulfide isomerase of the ER (PDI) [22] to molecular oxygen to oxidize PDI. If the machinery recognizes faulty disulfide bonds, glutathione (GSH) reduces disulfide bonds [23]. This way, the reduced glutathione/oxidized glutathione (GSSH) ratio is decreased.

Increased protein-folding load in the ER may result in the accumulation of ROS [1], and cells have evolved various mechanisms to limit overproduction of free radicals. Accordingly, PERK mediates phosphorylation of the nuclear factor and erythroid 2-like 2 (NFE2L2; NRF2) [24] to facilitate its accumulation in the nucleus to upregulate the expression of a set of oxidant-detoxifying and antioxidant enzymes. This holds, for example, for NAD(P)H–quinone oxidoreductase, heme-oxygenase 1 (HO-1), and glutathione S-transferase (GST) [25]. Nrf2-mediated HO-1 upregulation was found in sulforaphane-treated bladder cancer T24 cells [26]. More importantly, it has been shown that the ER stress-inducing chemical tunicamycin weakly induced ROS accumulation in PERK competent cells [27]. However, ROS accumulation was markedly enhanced in PERK-deficient cells. This ROS-mediated cell death was partly rescued by antioxidant *N*-acetyl-l-cysteine (NAC) [27], suggesting that oxidative stress contributes to PERK-mediated ER stress signaling. Next, we will discuss how ROS causes ER stress.

### Interplay of ROS and ER stress

It is noteworthy that PERK deficiency may suppress ROS-induced ER stress leading to apoptosis in cancer cells [27]. Stable transfection of shRNA–PERK in breast cancer MDA-MB468 cells impaired ROS-mediated ER stress induction. Surprisingly, PERK was noted to be a component of the protein network connecting ER to mitochondria [28, 29]. It has been shown experimentally that PERK+/+ murine embryonic fibroblast (MEF) cells had mitochondria interwoven with the ER network. However, in PERK−/− MEF cells, co-localization of mitochondria with ER was considerably lower [30].

Long-term ER stress may trigger two major pro-apoptotic pathways [31–34]. One involves c-Jun N-terminal kinase (JNK) and p38 mitogen-activated protein kinase (MAPK), while the other involves CHOP. Accumulation of IRE-mediated activation of JNK and p38 MAPK-mediated activation may lead to apoptosis [35, 36]. Other pro-apoptotic factors may be transcriptionally activated by CHOP [37, 38].

For the JNK pathway, an antimicrobial peptide pardaxin, synthesized by the red sea flatfish *Pardachirus marmoratus* [39], was used as an example. Pardaxin was reported to contribute to antiproliferation and inducible apoptosis in several cancer cells [40–42]. Both PERK activity and eIF2α were noted in pardaxin-treated HeLa cells [43]. The nuclear translocation of ATF6 also indicated that pardaxin induced ER stress in HeLa cells. Pardaxin also induced ROS production to activate AP-1/c-Jun and NAC can revert it. C-Jun was also demonstrated to be essential for apoptosis as caspase-3/-7 activity was inhibited by c-Jun small interfering RNA (siRNA) silenced cells. Therefore, it was concluded that both ER stress and ROS-induced c-Jun were activated and reciprocally regulated in pardaxin-treated cancer cells [43]. Similar ROS-inducible activation of JNK was noted in berberine-treated breast cancer MDA-MB-231 and MCF-7 cells [44]. It subsequently triggered mitochondrial membrane depolarization and led to apoptosis, although the role of ER stress in berberine treatment was not addressed.

Interestingly, SMIP004 (*N*-(4-butyl-2-methyl-phenyl) acetamide) was reported to induce the activation of eIF2α and IRE1 for apoptosis in prostate cancer cells and it was rescued by siRNA application to knockdown both of them [45]. SMIP004 was also demonstrated to trigger both JNK and p38 activity. Moreover, a decreased ratio of the reduced form of GSH to oxidized GSH and ROS accumulation indicated that SMIP004-induced apoptosis occurred through both ER stress mechanisms. Sarsasapogenin, isolated from the plant *Anemarrhena asphodeloides* Bunge, effectively induced apoptosis via the upregulation of CHOP in HeLa cells [46]. Moreover, sarsasapogenin-induced ROS generation was markedly inhibited upon NAC treatment, suggesting that drug-induced ROS mediates ER stress.

Synthetic polyphenol conjugate, (E)-3-(3,5-dimethoxyphenyl)-1-(2-methoxyphenyl)prop-2-en-1-one (DPP-23), also induces ROS-mediated apoptosis in several cancer cells, and tumor growth was reduced in mice xenografted with human colon cancer HCT116 cells [47]. Knockdown with siRNA IRE1α was reported to inhibit caspase-4 cleavage in DPP-23-treated human pancreatic cancer MIA PaCa-2 cells. Accordingly, UPR was involved in DPP-23-induced apoptosis. DPP-23 dose-responsively induced the ROS generation in MIA PaCa-2 cells but not for primary normal pancreatic cells. The oxidative stress role of DPP-23-induced apoptosis was further validated by the finding that NAC recovered DPP-23-induced GSH depletion in PaCa-2 cells. Therefore, DPP-23 displayed selective ROS generation in pancreatic cancer MIA PaCa-2 cells but not for normal pancreatic cells. It also demonstrated that oxidative stress is an upstream event of ER stress-mediated apoptosis [47].

Carnosic acid, isolated from extracts of rosemary, was noted to effectively enhance CHOP and ATF4 in renal carcinoma Caki cells [48]. ROS was also enhanced in carnosic acid-treated cancer cells, and NAC considerably reduced carnosic acid-induced CHOP and ATF4 expression. Bortezomib and dipyridamole worked with effective synergy to enhance ER stress in treated cancer cells [49]. Importantly, relieving ER stress by protein translation inhibitor cycloheximide impaired drug-induced apoptosis. In addition, bortezomib and dipyridamole treatment-depleted GSH and ROS levels were drastically enhanced in leukemia and lymphoma cells [49].

### Damage-associated molecular patterns

Stressed, injured, or dying cells release or flag certain molecules on their outer plasma membrane that are functionally not immunogenic within cells [50]. However, these molecules can initiate an immunological response if released extracellularly or displayed at the cell surface. These signals are termed damage-associated molecular patterns (DAMPs) [51]. Some DAMPs are passively released, namely high mobility group proteins B1 (HMGB1). In contrast, some are actively secreted like adenosine triphosphate (ATP) and others (calreticulin (CRT) and heat shock protein (HSP)-90), which appear on the cell surface or accumulate on the outer leaflet of the plasma membrane.

PERK induces phosphorylation of the eIF2α-facilitated shipping of CRT from ER to the Golgi apparatus [52]. Mechanistically, it has been shown that targeted inhibition of PERK and/or phosphatidylinositol-4,5-bisphosphate 3-kinase, catalytic subunit alpha (PIK3CA; PI3K)/v-akt murine thymoma viral oncogene homolog (AKT) dramatically reduced the CRT translocation [53]. For example, DNA-dependent protein kinase, a catalytic subunit (DNA-PKcs)-mediated phosphorylation of AKT, appeared to be necessary for the cell surface appearance or CRT. Wogonin (5,7-dihydroxy-8-methoxyflavone) was noted to effectively functionalize these pathways in gastric carcinoma MKN-45 cells [53]. Immunogenic cell death (ICD) is stress dependent and requires ROS-based ER stress [54]. Several sources indicated that ER stress and ROS synergistically activated danger signaling pathways that contributed to mobilization of DAMPs to the extracellular space [55]. Surprisingly, combining tunicamycin or thapsigargin with a chemotherapeutic drug may induce apoptosis with an immunogenic effect in nature as well [56]. It is concluded that ROS production and ER stress are critical for ICD, and simultaneous induction is vital to induce immunogenicity. ROS production and ER stress considerably enhanced different types of DAMP emission.

It has recently been convincingly demonstrated that ICD-associated immunogenicity is significantly increased when type II ICD inducers are used. Hypericin-based photodynamic therapy (PDT) is primarily a type II ICD inducer and operates through ROS-based ER stress. Mechanistically, it has been shown that hypericin is a photosensitizer that promotes substantially enhanced ROS generation upon excitation by specific wavelength thus resulting in a targeted ROS-based ER stress. Rapidly emerging evidence has shown that PDT induces ICD in cancer cells [54, 57]. Vaccination of C57BL/6 mice with PDT-treated Lewis lung carcinoma (LLC) alone or dendritic cells pulsed with PDT-treated LLCs revealed considerably improved immunological responses against Lewis lung carcinoma [58].

### TRAIL-induced signaling and ER stress

Tumor necrosis factor-related apoptosis-inducing ligand (TRAIL)-mediated signaling has emerged as one of the most extensively studied pathways reported to selectively induce apoptosis in cancer cells [59]. TRAIL signals intracellularly through death receptors which belong to the tumor necrosis factor receptor superfamily. Death receptors (DR4 and/or DR5) possess a cytoplasmic death domain (DD). Death-inducing signaling complex containing FADD and pro-caspase-8 appear at the death receptor. However, rapidly accumulating experimental evidence has also revealed that the expression and cell surface appearance of DRs is notably reduced in TRAIL-resistant cancer cells. Multiple signaling cascades have been suggested to modulate TRAIL-induced signaling. ER stress has also been noted to intricately stimulate DR5 expression in cancer cells. In accordance with this approach, various natural and synthetic agents have been shown to enhance expression of DR4 and DR5 in cancer cells. In the following section, we discuss the relevant literature highlighting natural agents reportedly involved in stimulating DR5 expression in cancer cells via the ER stress pathway.

PERK-eIF2α and ATF4-CHOP were notably enhanced in p53-deficient colorectal cancer cells treated with zerumbone (ZER) and celecoxib [60]. ROS scavengers drastically reduced CHOP expression. Interestingly, CHOP motif and ATF/cAMP response element motifs were identified at the proximal region of DR5 gene promoter. ATF3 depletion reduced DR5 expression and enforced expression of ATF3 significantly increased DR5 expression [60]. Histone deacetylase inhibitors (HDACi) also effectively induced activation of PERK and eIF2α in p53-deficient colorectal cancer cells [61]. Moreover, ATF4/ATF3/CHOP-mediated upregulation of DR5 was also notable in p53-deficient cancer cells. Agonistic anti-DR5 monoclonal antibody markedly enhanced apoptosis in cells treated with HDACi [61]. PKCδ is a member of the protein kinase C (PKC) family and undergoes proteolytic cleavage between catalytic domain and regulatory domain in stressed cells. CHOP and DR5 were not enhanced in ATF3- and ATF4-depleted cancer cells treated with bortezomib, a proteasome inhibitor [62]. It is also relevant to mention that MAPK–extracellular signal-regulated kinase (ERK) inhibitor, U0126, also impaired bortezomib-mediated increase in DR5. ATF4, ATF3, and CHOP were downregulated in PKCδ-deficient cancer cells [62].

Casticin, isolated from *Fructus viticis* and *Fructus monensin*, a polyether ionophore antibiotic enhanced DR5 via CHOP in different cancer cell lines [63, 64]. A recent report found ER stress responses in TRAIL-treated cancer cells including caspase-12 activation, while caspase-12 inhibition prevented apoptosis [65]. Na(+)-H(+) exchanger 1 (NHE1) from negatively regulating CHOP in cancer cells. NHE1 inhibitor cariporide-treated cells increased in the CHOP-mediated upregulation of DR5 [66]. Parthenolide, a sesquiterpene lactone, notably increased ATF4 and CHOP in lung cancer cells. Moreover, depletion of either ATF4 or CHOP severely abrogated the parthenolide-mediated upregulation of DR5 [67]. More importantly, JNK and p38 MAPK were also noted to be essential for stimulating expression of DR5 in cancer cells by tocotrienols [68]. Other drugs with similar effects for DR5-mediated ER stress pathways are summarized in Table 1.

**Table 1:** Natural products that mediate oxidative stress to enhance DR5 via the ER stress pathway in different cancer cell lines

Agents (sources)	Targets	Cancer type/cell lines	References
Verrucarin A (from several molds)	ROS↑	Liver cancer cells (TRAIL-resistant Hep3B cells)	[69]
	p-eIF2α↑; CHOP↑		
	DR5↑		
Guggulsterone (from *Commiphora mukul*)	ROS↑	Liver cancer cells (Hep3B; HepG2)	[70]
	p-eIF2α↑; CHOP↑		
	DR5↑		
Curcumin (from turmeric)	ROS↑	Liposarcoma cells (SW872)	[71, 72]
	CHOP↑; SERCA2↓		
	DR5↑		
5,7-Dimethoxyflavone (from *Leptospermum scoparium*)	ROS↑	Liver cancer cells (Hep3B, Huh-7, Hep G2)	[73]
	CHOP↑; GPR78↑; ATF4↑		
	DR5↑		

*SERCA2* sarcoplasmic/endoplasmic reticulum calcium ATPase 2

### Natural products with ROS and ER stress-modulating effects

Although many natural products have been reported to be ROS inducible [74–76], the role of ER stress was not emphasized. Recently, IRE1-mediated activation of JNK was found to be related to ER stress-induced apoptosis as evidenced by its pro-survival role during ER stress and the ability to reduce oxidative stress. JNK3 level was considerably higher in BH3 mimetic S1-treated ovarian cancer SKOV3/DDP cells. Targeted inhibition of JNK3 improved BH3 mimetic S1-induced apoptosis in cancer cells [77]. Peroxiredoxin 4 (PRDX4), an ROS-reducing enzyme, facilitates the appropriate folding of proteins in ER and frequently upregulates proteins in high-grade glioma cells [78]. Piperlongumine, a natural plant product effectively inhibited PRDX4 in glioma cells [79]. B cell-specific transcription factor (BACH2), a transcriptional repressor, has been reported to inhibit expression of antioxidant enzymes (superoxide dismutase and catalase) and antiapoptotic genes (BCL2, Bcl-xL, and MCL-1). Bortezomib has been shown to downregulate antioxidant and antiapoptotic genes by promoting nuclear accumulation of BACH2 in mantle cell lymphoma Jeko and SP53 cells [80].

PERK and IRE1 have been noted to be active in colon cancer SW480 cells treated with extracts of brown alga *Dictyopteris undulata* (DUE) [81]. Moreover, proteolytically processed ATF6 and CHOP were notably enhanced in DUE-treated cancer cells. Knockdown by siRNA CHOP can reduce DUE-induced apoptosis [81]. However, there is direct evidence suggesting differential mechanisms of action through which algal products exert influence. Fucoidan isolated from the marine brown alga *Fucus vesiculosus* has been shown to differentially modulate ER stress sensors [82]. For example, GRP78 was decreased in the fucoidan-treated MDA-MB-231 breast cancer cells. However, in HCT116 colon cancer cells, fucoidan treatment initially enhanced GRP78 expression, followed by a reduction of GRP78 with increasing fucoidan dosages. Moreover, phospho-IRE1 level was reduced and the generation of spliced X-box binding protein 1 splicing (XBP-1s) from unspliced XBP-1 mRNA is also reduced upon treatment. Interestingly, eIF2α phosphorylation and active eIF2α-mediated upregulation of CHOP were also noticed in breast cancer cells [82].

Sulforaphane, an isothiocyanate isolated from cruciferous vegetables, reportedly enhanced ER stress in terms of GRP78 and CHOP expression in bladder cancer T24 cells coupled with Nrf2-mediated oxidative stress and apoptosis [26]. Using hepatocellular carcinoma Hep3B cells, sulforaphane has also been reported to inhibit cell proliferation and telomerase activity involving oxidative stress [83]. Pretreatment of NAC can restore the inhibitory effects of hTERT expression and Akt phosphorylation by sulforaphane. These results suggest that sulforaphane-induced ROS generation may interplay with its ER stress effects. Other drugs with similar effects for ROS modulating effects to induce and inhibit ER stress pathways are summarized in Table 2.

**Table 2:** Natural products modulating ROS and ER stress activity in cancer and other cell lines

	Agents	Targets	Cancer and other cell lines	References
ER stress inducers	ω-Hydroxyundec-9-enoic acid (ω-HUA) (from wild rice (*Oryza officinalis*))	ROS↑	Lung cancer cells (H1299, A549, HCC827)	[84]
		CHOP↑		
	Cantharidin (from the insect *Mylabris phalerata* Pallas)	ROS	Lung cancer cells (H460)	[85]
		GRP78↑; IRE1α↑; IRE1β↑; ATF6α↑		
	Ampelopsin (from *Ampelopsis grossedentata*)	ROS	Breast cancer cells (MCF-7; MDA-MB-231)	[86]
		GRP78↑; p-PERK↑; p-elF2α↑		
		cleaved ATF6α↑; CHOP↑		
	Licochalcone A (from licorice *Glycyrrhiza inflate*)	ROS↑	Liver cancer cells (HepG2)	[87]
		CHOP↑		
	Isoliquiritigenin (from licorice *Glycyrrhiza glabra*)	ROS↑	Cervical cancer cells (HeLa)	[88]
		p-eIF2α↑; GRP78↑		
	Brefeldin A (BFA) (from *Penicillium brefeldianum*) [89]	ROS↑	Ovarian (OVCAR-3); lung (A549); colorectal (colo 205); breast (MDA-MB-231) cancer cells	[90–93]
		XBP1↑; GRP78↑		
		CHOP↑		
	Honokiol (HNK) (from *Magnolia obovata*) [94]	ROS↑	Chondrosarcoma (JJ012 and SW1353); gastric (AGS and MKN-45) cancer cells	[95–98]
		p-eIF2α↑; GRP78↑		
		CHOP↑		
	Delta(9)-tetrahydrocannabinol (THC) (from *Cannabis sativa*)	ROS ↑	Glioblastoma cells (SF126, U251, U87)	[99, 100]
		p-eIF2α↑		
	Resveratrol (from grapes) [101]	ROS↑	Colon (HT29); leukemia (K562); nasopharyngeal (NPC-TW076 and NPC-TW039); gastric (SGC7901); lung (A549) cancer cells	[102–106]
		XBP1↑; p-eIF2α↑; GRP78↑		
		CHOP↑		
	PABA/NO (from plant) [107]	ROS↑	Liver (HepG2); leukemia (HL60); ovarian (SKOV3) cancer cells	[108, 109]
		CHOP↑		
	Prodigiosin (from *Serratia marcescens*)	ROS↑	Pancreatic (8898); breast cancer cells (MCF-7 and MDA-MB-231)	[110, 111]
		p-eIF2α↑; PERK↑; GRP78↑; ATF6α↑		
		CHOP↑		
ER stress inhibitors	Benzodiazepines (from *Aspergillus ochraceus*)	ROS↓	Mesencephalic Progenitors (CSM14.1); neurons and neural stem cells; pheochromocytoma (PC12) cells	[112, 113]
		GRP78↓		
	Baicalein (from *Scutellaria baicalensis* Georgi) [114]	ROS↓	Neuronal HT22 cells; cardiomyocytes	[114, 115]
		CHOP↓		
	Cordycepin (3′-deoxyadenosin) (from *Cordyceps militaris*) [116]	ROS↓	Neuronal HT22 cells	[117]
		CHOP↓		
	Kifunensine mannosidase inhibitor (from *Kitasporia kifunensis*)	ER alpha-mannosidase↓	Endometrial stromal cells (HIESC); cervical cancer cells (HeLa)	[118, 119]
		CHOP↓		
	1-Deoxymannojirimycin hydrochloride (from *Lonchocarpus sericeus*)^a^	ER alpha-mannosidase↓	Pheochromocytoma (PC12) cells	[120]
		CHOP↓		

*PABA/NO O*(2)-[2,4-dinitro-5-(*N*-methyl-*N*-4-carboxyphenylamino)phenyl]1-(*N*,*N*-methylamino) diazen-1-ium-1,2-diolate; *prodigiosin* 2-methyl-3-pentyl-6-methoxyprodiginine

^a^This drug has a different reported effect that inhibition of alpha-mannosidase in liver cancer cells (7721) induces ER stress in terms of CHOP, XBP1, and GRP78 overexpressions [121]

## Conclusion

Accumulating evidence demonstrates how oxidative stress is generated from natural agents and synthetic chemicals that trigger ER stress-induced apoptosis. This is of particular interest for molecular oncologists. The functionalizing apoptotic machinery through different signaling pathways could be an effective approach in cancer treatment. ER stress-induced transcriptional upregulation of TRAIL receptors can be used to efficiently restore TRAIL-induced apoptosis in TRAIL-resistant cancers. Several drugs with ROS inductions and repressions have been suggested as being ER stress inducible and inhibitory. With ROS modulation, the ER stress effects of drugs were differentially modulated, making them more suitable for therapeutic purposes. The molecular approach needs additional investigation, and data obtained from preclinical studies will help us to identify natural products or synthetic chemicals with suitable therapeutic efficiency but minimal side effects. Pharmacokinetic studies will further shortlist candidates in the ever increasing list of chemopreventive agents.
